# Successful acclimatization of mandrills (*Mandrillus sphinx*) translocated to Conkouati-Douli National Park, Republic of Congo, as measured by fecal glucocorticoid metabolites

**DOI:** 10.1093/conphys/coad025

**Published:** 2023-06-08

**Authors:** Miles C Woodruff, Rebeca Atencia, Debby Cox, Glenn T Woodruff, Catharine J Wheaton, Shana R Lavin, Joanna M Setchell

**Affiliations:** Department of Anthropology, Durham University, South Road, Durham, DH1 3LE, UK; The Jane Goodall Institute, 1120 20th St NW, Washington, DC 20036, USA; The Jane Goodall Institute, 1120 20th St NW, Washington, DC 20036, USA; The Jane Goodall Institute, 1120 20th St NW, Washington, DC 20036, USA; Disney’s Animals, Science and Environment, Bay Lake, FL, 32830, USA; Disney’s Animals, Science and Environment, Bay Lake, FL, 32830, USA; Department of Anthropology, Durham University, South Road, Durham, DH1 3LE, UK

**Keywords:** welfare, translocation, reintroduction, pre-release enclosure, human-wildlife interactions, ethics

## Abstract

Translocation and reintroduction are common tools in conservation management and can be very successful. However, translocation can be stressful for the animals involved, and stress is implicated as a major cause of failure in release programs. Conservation managers should therefore seek to understand how the stages of translocation impact stress physiology in the animals involved. We quantified fecal glucocorticoid metabolites (fGCMs) as a noninvasive measure of response to potential stressors during a translocation of 15 mandrills (*Mandrillus sphinx*) into Conkouati-Douli National Park, Republic of Congo. The mandrills were initially housed in a sanctuary, transferred to a pre-release enclosure in the National Park and then released into the forest. We collected repeated fecal samples (n = 1101) from known individuals and quantified fGCMs using a previously validated enzyme immunoassay. Transfer from the sanctuary to the pre-release enclosure correlated with a significant 1.93-fold increase in fGCMs, suggesting that transfer was a stressor for the mandrills. fGCM values decreased over time in the pre-release enclosure, suggesting that the mandrills recovered from the transfer and acclimatized to the enclosure. Release to the forest was not linked to a significant increase in fGCMs over the final values in the enclosure. Following release, fGCMs continued to decrease, fell below sanctuary values after just over a month and were about half the sanctuary values after 1 year. Overall, our results suggest that the translocation, although initially presenting a physiological challenge to the animals, was not detrimental to the well-being of the animals over the timescale of the study and, in fact, may have been beneficial. Our findings show the value of non-invasive physiology in monitoring, evaluating and designing wildlife translocations and, ultimately, contributing to their success.

## Teaser

Stress is implicated as a major cause of failure in wildlife translocations. We monitored the stress response non-invasively during a mandrill release. Our findings suggest a pre-release enclosure allowed mandrills to recover from transfer and the mandrills adjusted well to the forest, showing the value of non-invasive physiology in translocations.

## Introduction

Translocations, in which humans move animals from the wild or captivity and release them into the wild, are commonly used in conservation, to restore extirpated populations (reintroduction), fortify depleted populations or restore ecosystem functions and processes (Seddon *et al* 2014). Humans also translocate animals for many other reasons, including to resolve conflicts over resources between humans and animals, to rescue animals when humans destroy their habitat, and in the belief that translocation improves the welfare of captive animals (e.g. primates: Beck, 2018). In all cases, we have a responsibility to maximize the well-being and survival of the translocated animals (Beck, 2016). However, despite efforts to improve evidence-based practice (e.g. IUCN/SSC, 2013) and increases in peer-reviewed studies of translocations (Seddon *et al.,* 2007), many such projects continue to suffer from low success, or unclear outcomes, due to poor planning, an absence of post-release monitoring and lack of transparency (Fischer and Lindenmayer, 2000; Germano *et al.,* 2014, 2015; Taylor *et al.,* 2017; Beck, 2018).

One factor thought to have a major influence on translocation success is stress (Teixeira *et al.,* 2007; Dickens *et al.,* 2010; Parker *et al.,* 2012), making it important to consider individual welfare in translocations (Harrington *et al.,* 2013). When an animal encounters a stressor, the stress response activates the hypothalamic–pituitary–adrenal axis, releasing physiological mediators, including glucocorticoids, which attempt to return the animal to homeostasis (Sapolsky *et al.,* 2000; Romero *et al.,* 2015). This is a normal, adaptive response; however, extreme or prolonged exposure to stressors can be dangerous. Stress and the stress response are often split into acute (shorter-term), which is considered adaptive, and chronic (longer-term), which is assumed to be deleterious, but the evidence underpinning this binary is not clearcut, particularly in wild animals (Romero *et al.,* 2009; Beehner and Bergman, 2017).

Stress is likely to be unavoidable in translocations, with potential stressors including rescue or capture, confinement, veterinary examinations, transportation to the release site, release, adaptation to new environment, other novel stimuli and the presence of humans during translocation and post-release monitoring (Teixeira *et al.,* 2007). Importantly, the effects of these multiple challenges on an individual may be cumulative, affecting survival and reproduction (Teixeira *et al.,* 2007). Evaluating the stress response across the phases of a translocation program can inform strategies to mitigate these effects (Teixeira *et al.,* 2007). However, a 2013 systematic review of animal reintroductions found that only 2% monitored stress levels (Harrington *et al.,* 2013). Almost a decade on, our understanding of stress in translocations remains poor, despite acknowledgement of the importance of animal welfare in translocation success (Parker *et al.,* 2015) and a call to include physiology more generally in conservation translocations (Tarszisz *et al.,* 2014).

Existing studies of the stress response during translocations in mammals focus on translocation of wild animals (Aguilar-Cucurachi *et al.,* 2019, Batson *et al.,* 2017, Franceschini *et al.,* 2008, Turner *et al.,* 2002, Wells *et al.,* 2004, Taylor *et al*., 2016). Wild animals are likely to require little acclimatization to a new environment, and instead, the focus is on minimizing time in captivity, because it is likely to be stressful for wild animals. However, primates are often in a very different situation.

Habitat destruction, hunting and the pet trade have created an abundance of confiscated primates housed in sanctuaries (Trayford and Farmer, 2013). Such sanctuaries often have the goal of releasing primates into the wild, motivated by limited space and the belief that translocation improves primate welfare (Faust *et al.,* 2011; Beck, 2018). The value of such welfare-motivated primate translocations is hotly debated (Palmer, 2000; Beck, 2018). If an animal is translocated under appropriate circumstances and adapts to life in the wild, its well-being may improve. However, many animals suffer and die as the direct outcome of translocation, and it is unclear whether release truly benefits the well-being of individual primates (King *et al.,* 2011; Beck, 2018; Guy *et al.,* 2021). Scientific studies of welfare-related translocations (e.g. Rodriguez-Luna and Cortés-Ortiz, 1994; Strum, 2005) can inform future conservation efforts, which also require good animal welfare to be successful (Guy *et al.,* 2014; Beck, 2016).

The question of how to improve welfare, and therefore survival, in primate translocations is becoming urgent, with populations of approximately 70% of primate species in decline (Estrada *et al.,* 2017) and translocations likely to become increasingly important in the survival of the remaining populations. However, the Conservation Evidence website (Junker *et al.,* 2020) lists all actions related to rehabilitation and reintroduction for primates as ‘unknown effectiveness (limited evidence)’, although ‘reintroduce primates into habitats where species is absent’ is listed as ‘likely to be beneficial’. To our knowledge, no study has yet examined the stress response during translocation in rehabilitated primates, despite the importance of such studies in informing translocation strategies. We used a previously-validated non-invasive and field-friendly methods to measure fecal glucocorticoid metabolites (fGCMs) in mandrills (*Mandrillus sphinx*) (Lavin *et al.,* 2019) at each stage of a translocation: mandrills housed at the sanctuary, transferred to a pre-release enclosure and released into the forest. fGCMs are a popular physiological biomarker of the stress response, because they can be measured non-invasively and provide an integrated measure of glucocorticoid activity over a period of hours or days, in contrast to the large daily fluctuations in blood saliva or urine samples (Whitten *et al.,* 1998; Hodges and Heistermann, 2011). We tested predictions arising from five hypotheses (Table 1). We also explored sex differences in response to the release process because females and males may respond differently to release.

## Materials and Methods

### Study site and subjects

We conducted our study at three sites: the mandrill enclosures at Tchimpounga Sanctuary in southern Congo (UTM 32M 814303 9 500 175) (the sanctuary); a pre-release enclosure in Conkouati-Douli National Park (UTM 32M 774300 9 567 971); and the forest surrounding the pre-release enclosure, into which the animals were released.

Mandrills are large, sexually dimorphic, forest-dwelling, semi-terrestrial, social primates, found in Cameroon, Equatorial Guinea, Gabon and Republic of Congo (Grubb, 1973). They are listed as Vulnerable by the IUCN, and the most immediate threat to their survival as a species is hunting for meat (Abernethy and Maisels, 2019). The rescued mandrills we studied were orphaned by the trade in wild meat and confiscated by, or with the approval of, the Congolese Ministère de l’Economie Forestière. They were transferred to Tchimpounga where they were quarantined for at least 30 days, screened for communicable diseases and treated if necessary. They were then socialized and housed in groups.

At the beginning of the study, Tchimpounga was responsible for 16 confiscated mandrills (10 males, 6 females). Release Groups 1 and 2 were composed of 10 of these 16 mandrills, aged approximately 4 to 11 years old who had been housed together in a stable group for over a year and were considered appropriate for release by their caretakers (Table 2). Group 3 comprised five animals that arrived at the sanctuary between August 2013 and November 2014 and were considered appropriate for release.

**Table 1:** Hypotheses and predictions tested in a study of fecal glucocorticoid metabolite (fGCM) values^a^ in mandrills released into Conkouati-Douli National Park, Republic of Congo, in 2013–15

Number	Hypothesis	Prediction	Test	Supported?
1	Events associated with transfer are stressors for the mandrills	Transfer to pre-release enclosure correlates with an increase in FGCM values compared to values at the sanctuary	Compared fGCM values during the final month in the sanctuary with values during the first week in the pre-release enclosure	Yes
2	It takes time for the mandrills to acclimatize to the pre-release enclosure	FGCM values decrease over time in the pre-release enclosure	Tested for a relationship between FGCM values and time (days) in pre-release enclosure^b^	Yes
3	Release from the pre-release enclosure is a stressor for the mandrills	Release correlates with an increase in FGCM values	Compared fGCM values during the last month in pre-release enclosure with values for first week post-release. Excluded samples from the first 30 days in the enclosure to allow for acclimatization.	No
4	It takes time for the mandrills to acclimatize to the release environment	FGCM values decrease over time after release	Tested for a relationship between FGCM values and time (days) post-release	Yes
5	The forest is less stressful for the mandrills than the captive environments	FGCM values are lower post-release than in the sanctuary or the pre-release enclosure	Compared all available fGCM values across the three environments.	Partly

a ^a^FGCM values measured using a validated 11-β-hydroxyetiocholanolone enzyme immunoassay. Glucocorticoids are metabolic hormones associated with the stress response.

b ^b^Data up to 196 days, after which only one animal was sampled.

**Table 2:** Study subjects, days at each stage and numbers of fecal samples collected in a study of mandrills reintroduced into Conkouati-Douli National Park, Republic of Congo, in 2013–15

ID (Code)	Date arrived at Tchimpounga	Group	Sex	Age-class	Collar	Days in each stage during the study (number of samples)			
						Sanctuary^a^	Pre-release enclosure	Post-release	Total
George (GEO)	29 Sept 2008	1	F	Adult	GPS	88 (16)	197 (49)	5 (1)^b^	290 (66)
Dominique (DOM)^c^	28 July 2010	1	F	Adult	GPS	88 (26)	197 (56)	362 (56)	647 (138)
Kiki Mpaka (KM)	10 Nov 2008	1	M	Adult	Argos	88 (12)	197 (46)	362 (105)	647 (164)
Obia (OB)	7 Nov 2008	1	M	Adolescent	GPS	88 (22)	197 (61)	362 (56)	647 (164)
Madol (MAD)	2012^d^	1	M	Adolescent	GPS	88 (19)	197 (56)	362 (84)	647 (139)
Kiki Tchiali (TCH)	10 Nov 2008	2	M	Adolescent		88 (24)	17 (7)	0 (0)^b^	105 (139)
Gagaga (GAG)	26 Mar 2009	2	M	Adolescent	Argos	88 (15)	21 (10)	57 (26)^b^	166 (51)
Gayard (GAY)	13 July 2012	2	M	Adolescent		0 (0)	21 (7)	355 (127)	376 (134)
Mobote (MOB)^c^	14 June 2012	2	F	Adolescent		0 (0)	19 (3)	355 (55)	374 (58)
Vue de Loin (VDL)	24 April 2012	2	M	Adolescent	GPS	0 (0)	19 (10)	33 (88)	374 (98)
Suzo (SUZ)	4 April 2014	3	M	Juvenile		0 (0)	76 (18)	33 (2)	109 (20)
Nzelly (NZ)	11 Sept 2013	3	F	Juvenile		0 (0)	76 (13)	33 (3)	109 (26)
Kento (KEN)	6 Nov 2014	3	F	Juvenile		0 (0)	57 (2)	33 (6)	81 (8)
Brek (BRK)	28 Sept 2014	3	M	Juvenile		0 (0)	57 (8)	33 (12)	81 (20)
Egeuo (EGO)	11 Sept 2013	3	M	Juvenile		0 (0)	57 (15)	33 (16)	90 (31)
Mean						41 (10)	94 (24)	161 (42)	316 (75)
SD						45 (11)	78 (22)	168 (43)	234 (55)

a ^a^Staff were in the forest with the released animals and not available to collect samples from some of the animals in Group 2 or any of the animals in Group 3 while they were at the Tchimpounga Sanctuary.

b ^b^Animals that were subsequently captured and taken back to the sanctuary because they did not adapt well to release.

c ^c^These two females conceived during the study and gave birth post-release

d ^d^Full date unknown

**Table 3:** Results of General Linear Mixed Models testing predictions related to fecal glucocorticoid metabolite values in mandrills released into Conkouati-Douli National Park, Republic of Congo, in 2013–15

Prediction	Parameter	Estimate	SE	df	t	p	95% CI	
							lower	upper
1	Intercept	2.855	0.133	83	21.49	<0.001	2.591	3.120
	Sanctuary^a^ vs. pre-release enclosure^b^	−0.287	0.051	70.66	−5.63	<0.001	−0.388	−0.185
	Female vs male^b^	0.062	0.067	6.06	0.93	0.390	−0.101	0.225
2	Intercept	2.777	0.163	346	17.00	<0.001	2.456	3.099
	Days in pre-release enclosure	−0.001	0.000	340.20	−4.91	<0.001	−0.002	−0.001
	Female vs male^b^	0.012	0.075	10.46	0.15	0.880	−0.155	0.178
3	Intercept	2.707	0.160	107	16.90	<0.001	2.390	3.024
	Final month pre-release vs week 1 in wild^b^	−0.095	0.060	103.60	−1.58	0.117	−0.214	0.024
	Female vs male^b^	0.062	0.094	10.36	0.65	0.526	−0.147	0.271
4	Intercept	2.634	0.168	340.17	15.65	<0.001	2.303	2.966
	Days after release	−0.001	0.000	498.82	−6.06	<0.001	−0.001	0.000
	Female vs male^b^	0.097	0.123	11.25	0.79	0.448	−0.173	0.367
5	Intercept	2.602	0.163	982	15.99	<0.001	2.282	2.921
	Sanctuary^a^ vs. forest^b^	0.066	0.029	901.47	2.31	0.021	0.010	0.122
	Pre-release enclosure vs. forest^b^	0.084	0.020	963.06	4.21	<0.001	0.045	0.123
	Female vs male^b^	0.056	0.064	11.70	0.87	0.399	−0.084	0.196

a ^a^Sanctuary values are adjusted to account for the effect of faster drying time on fGCM values (Lavin *et al.,* 2019).

b ^b^Reference group

### The release process and enclosures

We followed the IUCN Guidelines for Nonhuman Primate Reintroductions (Baker, 2002) as far as possible. We worked closely with governmental and local authorities during the preparation process and release. We selected the release location based on findings from a survey of the release area (Woodruff, 2019) and constructed a pre-release enclosure in the chosen area.

At Tchimpounga, the mandrills were housed in two outdoor enclosures (30 m^2 ^and 43 m^2^) with corrugated tin roofs, walls 3 m high, chain-link sides, dirt floors and a 1 m concrete brick foundation around the perimeter (**Online Appendix A**). A third enclosure (43 m^2^) was built after Group 1 was transferred to the pre-release enclosure. Staff fed the mandrills with approximately 2 kg per animal per day of leafy greens, fresh branches, tree limbs, entire *Aframomum* plants and cooked rice.

We constructed a pre-release enclosure (~58 m^2^) at the release site in a similar fashion to the Tchimpounga enclosures (**Online Appendix A**). Mandrills in the pre-release enclosure had the same diet as at Tchimpounga, to aid their transition and avoid large differences in dietary fiber conditions, which can affect fGCMs (von der Ohe *et al.,* 2004).

We transferred Group 1 to the pre-release enclosure in August 2013, Group 2 in February 2014 and Group 3 in November/December 2014 (Fig S4**, Online Appendix B**). We sedated the animals by administering 0.05 mg/kg medetomidine and 5 mg/kg ketamine using a blowgun. Vets performed a final health check, placed the animals in separate pet carriers or crates for transport, then gave them the antidote Antisedan. Animals were awake for transport by truck (3–4 hours), then by boat (~30 minutes), and finally by hand (2 minutes) to the pre-release enclosure. Groups 1 and 2 spent 12 to 14 days together in the pre-release enclosure.

Prior to release, we fitted seven mandrills with mock collars in Jan 2014, then with tracking collars in Feb 2014 (Table 2). Each collar was less than 5% of the animal’s body mass (mean, 3.4%; range, 1.7–4.7%), in accordance with the standard set by the American Society of Mammologists (1998).

Circumstances beyond our control delayed the release of Group 1 for several months. We released Group 1 (n = 5) on 5 March 2014, after 197 days in the pre-release enclosure (Table 2). One female was recaptured and taken back to Tchimpounga because she was aggressive to observers. Group 1 remained near the pre-release enclosure after release, so we released Group 2 (n = 5) to join them 7 days later, meaning that Group 2 spent only 17–19 days in the pre-release enclosure. We returned one male to the sanctuary following a fight. We allowed Groups 1 and 2 access to the pre-release enclosure for one week after Group 2 was released. The two groups merged, except for an adolescent male who travelled a long way, and was recaptured and returned to the sanctuary because he was very thin (he survived). We released Group 3 (n = 5) 10 months later, after 57 to 76 days in the pre-release enclosure. Group 3 had become familiar with the combined Groups 1 and 2 while in the pre-release enclosure and had merged fully with them by 1 week after release.

After release, the mandrills used the forest surrounding the enclosure freely but remained near the enclosure. In the mornings and evenings, we led them away from the release site with ~ 2 kg of supplementary food each. We decreased this food in 10% increments based on the animals' condition and behavior. The animals were still receiving a cup of cooked rice daily at the end of the study (March 2015).

We did not remove the tracking collars at the end of the study to facilitate ongoing monitoring. Instead, we trained animals wearing collars to enter and exit the pre-release enclosure so that they could be sedated safely to remove their collars if their automatic drop-off mechanism failed.

Overall, of 15 mandrills, three were returned to Tchimpounga because their release was not successful, none died during the study and two infants were born, both of which survived.

### Fecal sampling and processing

We collected 1143 fecal samples between 22 May 2013 and 4 March 2015, with a mean of 73 (SD, 56; range, 8–156) samples per individual (Table 2). Samples were not evenly distributed across the study and among the mandrills for logistical reasons.

We collected fecal samples opportunistically from identified individuals. Previous studies of mandrills (Setchell *et al.,* 2008) found no diurnal variation in fGCMs so we collected samples throughout the day. We processed the samples using a validated field-friendly method for hormone extraction in this species (Lavin *et al.,* 2019). We discarded samples contaminated with urine. We removed debris from the exterior, homogenized the sample with a fresh stick, weighed 1 g of feces and placed this subsample in a 15 ml polypropylene tube with 10 ml 90% ethanol within 5 minutes. We shook the sample by hand for 5 minutes then let it rest on a table for a minimum of 4 hours before transferring 1 ml of the supernatant to an Eppendorf tube. At the sanctuary, we dried samples in a yogurt cooker. In the forest, we dried samples in an aluminum Dutch oven using a gas stove. We dried samples fully.

We shipped the dried samples to Disney’s Animal Kingdom® in March 2015, and stored them at −80°C until analysis, which we conducted in March to July 2015.

### Laboratory analysis

We used an enzyme immunoassay for 11β-hydroxyetiocholanolone (lab code 69a) that we have previously validated biochemically and biologically as a measure of the fGCM response in mandrills (Lavin *et al.,* 2019). We reconstituted dried samples in 0.5 ml methanol. 69a concentrations in serial dilutions of pooled mandrill fecal extracts (including a male pool, female pools, a high concentration pool and a low concentration pool) were parallel to the standard curve, based on visual inspection and a linear regression of the percent binding of the labelled 69a in the 69a assay including the interaction of the log of the standard concentration or log of the inverse of the sample dilution*category of standard versus sample (interaction term P > 0.05 indicating that the lines do not intersect; Systat 13). Based on these serial dilutions, we diluted samples (1:4–1:25; depending on concentration) to produce sample binding within the conservative readable range of the assay (20–80%). The average %B was 56% (±14% SD).

Coefficients of variation for intra-assay controls were < 20%, and inter-assay controls (n = 53, 96-well plates) were 8.72% for high and 14.25% for low concentration standards diluted with assay buffer and 16.69% for the high and 15.08% for the low concentration biological control (pooled mandrill fecal extracts). The mean CV for all samples run in duplicate was 6 ± 5% (SD). Assay sensitivity was 32 pg/well (90% binding). Exogenous 69a added to mandrill fecal extract yielded 98 ± 1.5% spike recovery.

### Statistical analysis

We used linear mixed effect models in IBM SPSS Statistics for Macintosh, Version 28.0 to test our predictions. We log10 transformed the fGCM values prior to analysis to achieve normally distributed residuals. In addition to our predictor variables (Table 1), we included sex as a fixed factor and subject ID as a random factor (intercept and slope) to account for repeated measures from individual animals. We did not include further explanatory variables, such as age, due to the small number of individuals contributing to the data set.

To report effect sizes, we back-transformed logged values to give fGCM values in ng/g wet feces and report the final value divided by the initial value. This fold change describes how much fGCM values change between the first and second measurement and is more informative than the difference. For example, a 2-fold change is a doubling, and a 0.5-fold change is half.

Drying time affected measurement of fecal 69a in our validation tests (Lavin *et al.,* 2019). Fecal extraction of 69a decreased by 2.8% between 4 and 24 hours, increased 36.7% between 24 and 48 hours, then increased only 4.5% between 48 and 96 hours. All samples at the sanctuary dried in < 24 hours, but samples in the forest took longer. To adjust for their faster drying time, we multiplied the fGCM value for sanctuary samples by 1.367, to reflect the increase in extraction that occurs between 24 and 48 hours. We present both adjusted and non-adjusted data where relevant.

We set alpha at 0.05 in all analyses. 

### Ethical note

This study received approval from the Animal Welfare Ethical Review Board at Durham University and permission from the Conservateur of the National Park to conduct the study and export samples for analysis. All transfers of hormone extracts followed international CITES regulations (CITES permit number: 0111125666). We only sedated animals to perform health checks, for transfer to the release site, to fit collars and to return some individuals to the sanctuary. All sedation was conducted by a qualified veterinarian or nurse.

## Results

### Prediction 1: Transfer to the pre-release enclosure correlates with an increase in fGCM values.

**Figure 1:**
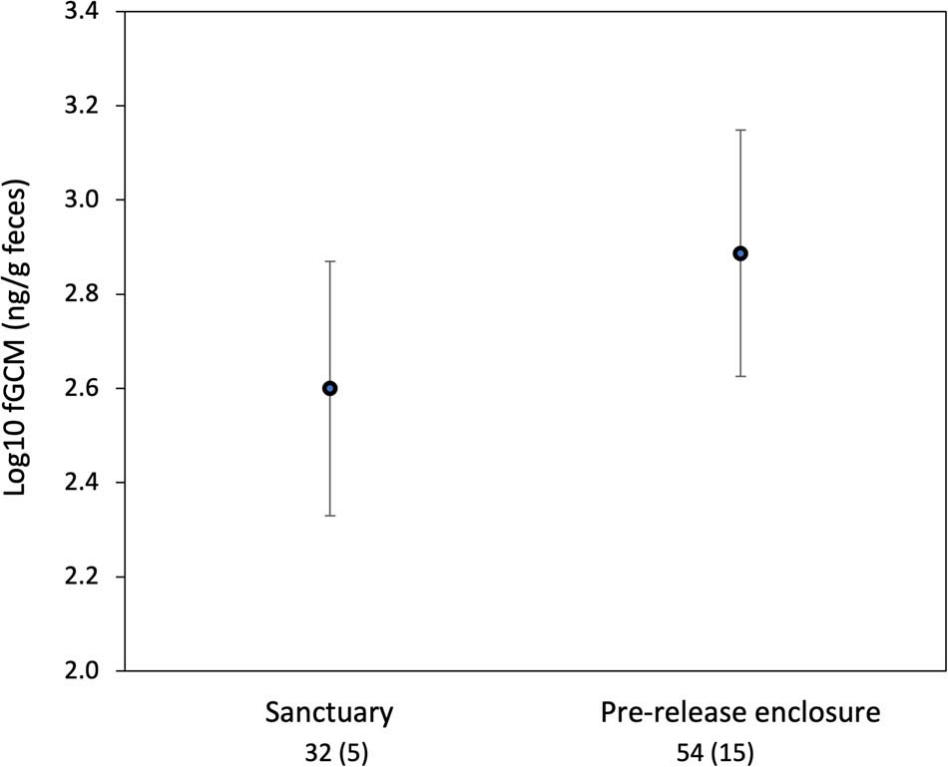
Mean log ± 95% CI fecal glucocorticoid metabolite (fGCM) values in mandrills during their last month in Tchimpounga sanctuary and their first week in a pre-release enclosure in Conkouati-Douli National Park, Republic of Congo, in 2013–15. Numbers below x axis show number of samples (number of animals). Sanctuary values are multiplied by 1.367 to account for the effect of differences in drying time (see methods). Mandrills were transferred in small groups at different times, so days since transfer do not correspond to the same calendar dates across individuals.

We found evidence to support Prediction 1. fGCM values increased significantly when mandrills were transferred from the sanctuary (adjusted values) to the pre-release enclosure (Table 3**,**Figure 1). Back-transforming the mean logged values showed a 1.93-fold increase in fGCMs from the last month in Tchimpounga sanctuary (398.1 ng/g) and the first week in the pre-release enclosure (769.1 ng/g). Unadjusted values showed a similar pattern (Figure S5**, Online Appendix B**), with a 1.6-fold increase. There was no significant influence of sex (Table 3).

### Prediction 2: fGCM values decrease over time in the pre-release enclosure

**Figure 2:**
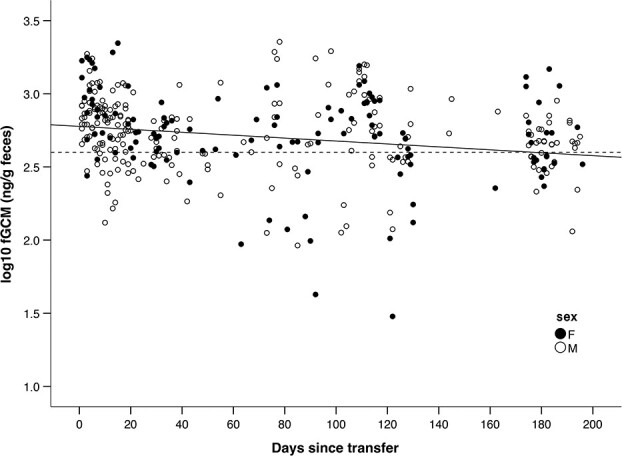
Log fecal glucocorticoid metabolite (fGCM) values in mandrills after transfer to a pre-release enclosure in Conkouati-Douli National Park, Republic of Congo, in 2013–2015. Data are for 351 samples from 15 mandrills. Fitted line is based on a mixed model accounting for ID as a random factor (y = −0.001x + 2.777). Dashed line indicates the mean value during the last month in the sanctuary. Mandrills were transferred in small groups at different times, so days since transfer do not correspond to the same calendar dates across individuals.

We found evidence to support Prediction 2. fGCM values decreased significantly over time in the pre-release enclosure (Table 3), with a great deal of variation (Figure 2). Based on back-transformed values, fGCM values decreased 0.64-fold over 196 days from the beginning of the time in the pre-release enclosure (598.4 ng/g) to the end (381.1 ng/g). By the end of the maximum time spent in the pre-release enclosure (196 days), the back-transformed fitted value (381.1 ng/g) was 0.96-fold lower than the mean fGCM values during the last month in the sanctuary (398.1 ng/g). There was no significant sex difference (Table 3).

Inspection of the plot of fGCMS over time in the pre-release enclosure suggested that the slope was higher during the first few weeks (Figure 2)*.* Post-hoc analyses using only data for the first 30 days showed a significant slope of −0.010 log(ng/g) or 0.98 ng/g per day (Supplementary Table S1**, Online Appendix B**), with a 0.50-fold decrease from day 0 (737.9 ng/g) to day 30 (369.8 ng/g) based on back-transformed values. Beyond 31 days, there was no significant relationship between fGCMs and time since transfer (Supplementary Table S1).

### Prediction 3: Release will correlate with an increase in fGCM values

**Figure 3:**
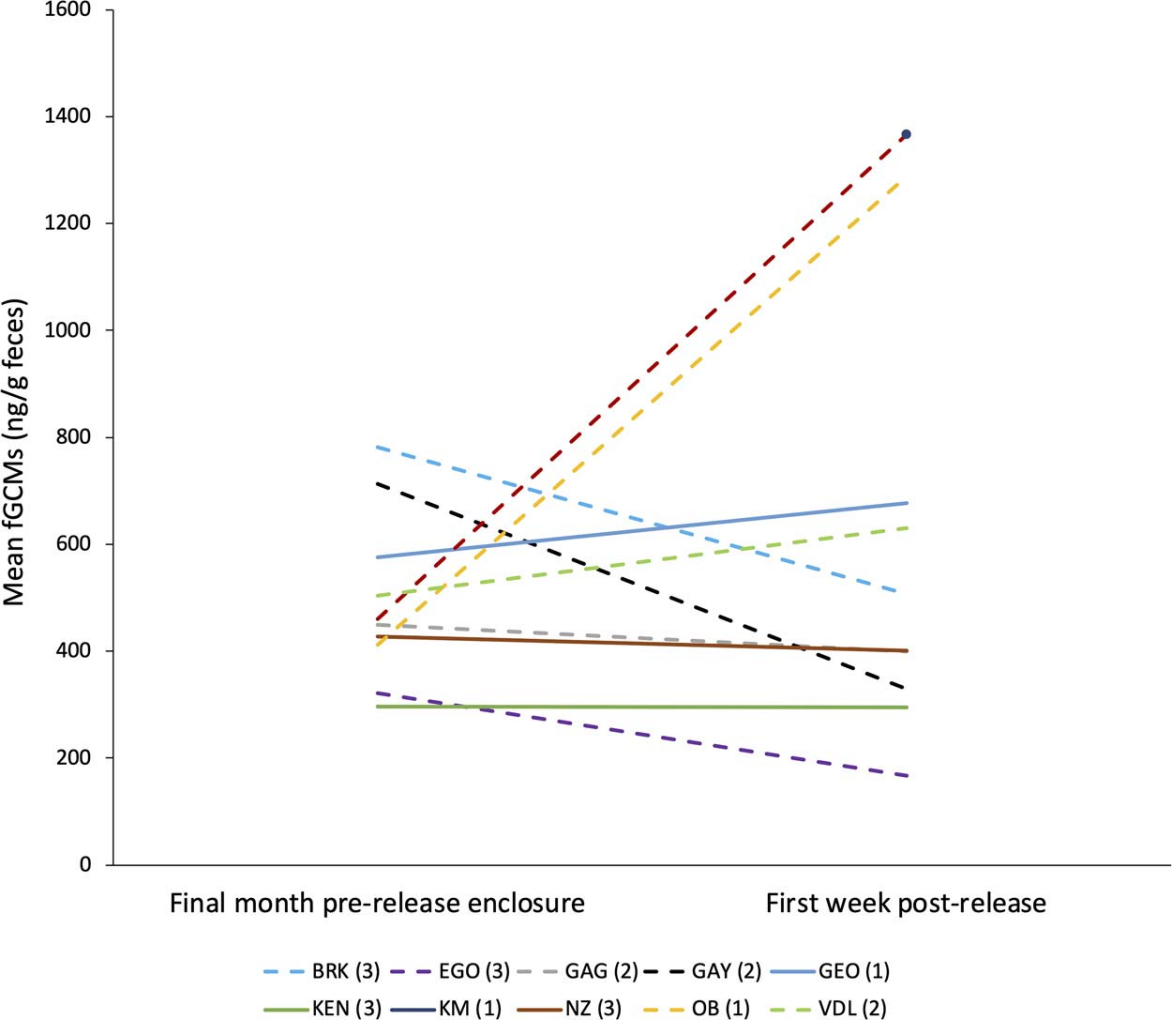
Mean fecal glucocorticoid metabolite (fGCM) values in mandrills during their last month in a pre-release enclosure in Conkouati-Douli National Park, Republic of Congo, and their first week post-release, in 2014–2015. Lines link the mean for each individual in the two conditions. Full lines are females, dashed lines are males. Numbers after ID codes indicate the release group.

We found no statistical support for Prediction 3. fGCM values during the first week after release were not significantly different to values in the pre-release enclosure, excluding the first 30 days in the pre-release enclosure to allow for acclimatization (Table 3). Values tripled for two males in Group 1, increased 1.2-fold for two animals and decreased 0.5- to 0.9-fold for the other five animals for whom we had paired data (Figure 3**, Online Appendix B**). There was no significant effect of sex (Table 3).

### Prediction 4: The fGCM response associated with release will decrease over time

**Figure 4:**
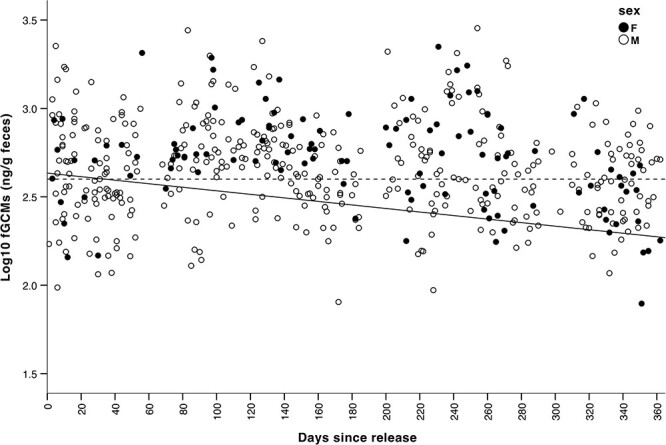
Log fecal glucocorticoid metabolite (fGCM) values  in mandrills during 1 year after release into Conkouati-Douli National Park, Republic of Congo, in 2013–15. Data are from 500 samples for 13 mandrills. Full line is based on a linear mixed model accounting for ID as a random factor (y = −0.001x + 2.634). Dashed line indicates the mean value during the last month in the sanctuary. Mandrills were transferred in small groups at different times, so days since transfer do not correspond to the same calendar dates across individuals.

We found statistical support for Prediction 4. Mean fGCM values decreased significantly over time, with no significant influence of sex (Table 3). The estimated effect of time since release on fGCM values was the same as that for time in the pre-release enclosure (Table 3), with back-transformed values showing a 0.43-fold reduction over 1 year (from 430.5 ng/g to 185.8 ng/g). Variation was high across the year (Figure 4). The fitted line crossed the mean for the final month in the sanctuary (adjusted values) after 34 days, and the back-transformed final fitted value at 365 days (185.8 ng/g) was 0.47-fold lower than the back-transformed mean during the final month at the sanctuary (398.1 using adjusted values, 0.64-fold lower than unadjusted values).

### Prediction 5: fGCM values are lower post-release than in the sanctuary or the pre-release enclosure

**Figure 5:**
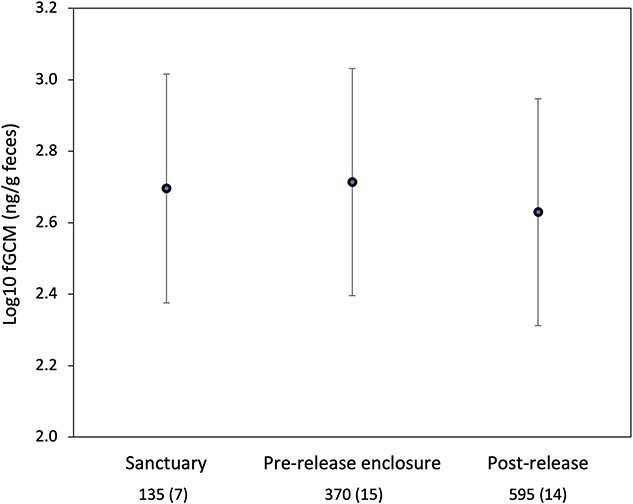
Mean log ± 95% CI fecal glucocorticoid metabolite (fGCM) values  in mandrills housed in Tchimpounga sanctuary, a pre-release enclosure and after release into Conkouati-Douli National Park, Republic of Congo, in 2013–15. Numbers below x axis show number of samples (number of animals). Sanctuary values are multiplied by 1.367 to account for the effect of differences in drying time (see methods).

Support for Prediction 5 depended on whether we adjusted the fGCM values at the sanctuary to account for differences in drying time. Analysis with adjusted values showed a significant difference between environments (Table 3), and post-hoc tests showed that this significant difference lies between values in the wild and the other two environments (pre-release p < 0.001, sanctuary p = 0.017), with no significant difference between values in the sanctuary and the pre-release enclosure (p = 0.533). Overall, back-transformed mean fGCM values increased 1.04-fold from the sanctuary (496.6 ng/g) to the pre-release enclosure (517.6n/g), decreased 0.82-fold from the pre-release enclosure to post-release (426.6) and decreased 0.86-fold from sanctuary to post-release values. Variation was high across all conditions (Figure 5).

The same analysis using unadjusted fGCM values also showed a significant difference across environments (Supplementary Table S1), with all pairwise comparisons significant (p < 0.05). Unadjusted sanctuary values were lower than in the other two environments (Figure S6**, Online Appendix B**).

## Discussion

We successfully monitored the fGCM response of a group of mandrills to a soft release into Conkouati-Douli National Park, Republic of Congo. We must exercise caution in interpreting fGCM concentrations in terms of how animals cope with stressors and whether the stressors are harmful to the animals and affect their survival (Romero *et al.,* 2015; Beehner and Bergman, 2017). However, even a mild response to a stressor (measured here as an increase in fGCMs) diverts resources away from other biological functions, reducing an animal’s ability to respond to other stressors.

Like many primate translocations (Beck, 2018), this project was primarily motivated by improving primate welfare. Mandrills are not yet in need of population reinforcement. However, studies of such translocations have crucial implications for conservation translocations, which also need animals to thrive (Harrington *et al.,* 2013). Below, we discuss the limitations of the study, then the implications of our findings for each stage of the translocation, before drawing more general conclusions concerning animal well-being and translocations. We provide specific discussion of mandrill translocation strategies in **Online Appendix C**.

### Limitations of the Study

Our study design was constrained by logistics. We were unable to sample all animals under all conditions, and the time animals spent in the different conditions varied greatly. Their diet also changed gradually over time post-release, as they consumed more natural forage, which may influence gut transit time, and therefore fGCM values. Moreover, the animals were not selected at random, but were chosen as suitable for translocation. As the study progressed, removal of animals that did not adapt well biases our analyses to those that did, and we have no controls with whom to compare the study animals. Our sample size was also constrained by the number of animals suitable for release and precluded analyses of factors which may explain some of the large variation in fGCMs between and within individuals across the whole translocation process. We did test the effect of sex, but although we found no sex differences in fGCM values in our analyses, we cannot draw strong conclusions about the influence of sex from our limited sample size.

### Transfer to the Pre-Release Enclosure

Transfer from the sanctuary to the pre-release enclosure correlated with a 1.93-fold increase in fGCMs (correcting sanctuary values for the effect of drying time). This supports Hypothesis 1, that events associated with transfer from the sanctuary to the pre-release enclosure, including anesthesia, transport and an unfamiliar enclosure) are stressors for the mandrills (Table 1). The pattern we observed in mandrills is similar to findings for wild zebras (*Equus grevyi*, Franceschini *et al.,* 2008), rhinoceros (*Ceratotherium simum*, *Diceros bicornis*,* *Turner *et al.,* 2002) and cheetahs (*Acinonyx jubatus*, Wells *et al.,* 2004), which all showed elevated fGCM values after transfer to a new location. Together these results suggest that the challenges of transfer and associated procedures induce a physiological response in the animals. If release is also stressful, then animals released before they have recovered from transfer may suffer cumulative effects, making them vulnerable to the potential negative side effects associated with homeostatic overload (Romero *et al.,* 2009). Thus, practitioners wishing to reduce the effects of translocation on animals should take measures to help animals recover safely from the stress of transfer prior to exposing them to any additional possible stressors. In this case, we used a pre-release enclosure, but this may not be appropriate for wild animals that are not already habituated to confined living conditions.

### The Pre-Release Enclosure

The IUCN recommends that captive primates are held in a pre-release enclosure, as part of a gradual transition to life in the wild (a ‘soft’ release) (Baker, 2002). However, this advice is not always followed, and where it is, the time spent in transfer enclosures and pre-release enclosures varies greatly and is based largely on the opinions of those conducting the release (Guy *et al.,* 2013) and financial and logistical constraints, rather than on empirical studies. We provide rare empirical data on the relationship between time spent in the pre-release enclosure and fGCMs, as a measure of physiological acclimatization. fGCM values decreased over time in the enclosure, suggesting that it allowed the mandrills to recover from the transfer and acclimatize to the forest environment. The decrease in fGCMs was greatest in the first month (0.50-fold decrease) after which there was no significant relationship between time in the pre-release enclosure and fGCMs, suggesting that the first month, when the mandrills were recovering from the transfer, was most important.

An alternative explanation for the decrease in fGCM values over time in the pre-release enclosure is that the animals remained chronically stressed, but their hormonal response decreased over time (Cyr and Romero, 2009). For example, decreasing and low fecal GCs when wild rhinoceros were held captive were associated with suppressed reproductive steroids, suggesting that the low GCs were due to suppression of HPA activity in response to severe or prolonged stress, rather than acclimatization (Linklater *et al.,* 2010). However, behaviors including teeth-grinding in the dominant male and alarm calling by all animals also reduced over time in the mandrills (MW, unpublished data), suggesting that the reduction in their fGCMs is due to decreased stress, rather than a reduced ability to respond to that stress or a state of adrenal exhaustion in which it fails to respond at all (Selye, 1946; Sayers, 1950; Johnstone *et al.,* 2012). Moreover, females showed ovarian cycles, and one conceived in the pre-release enclosure (**Online Appendix D**), suggesting that the stress response to transfer and translocation to the pre-release enclosure was not of sufficient magnitude or duration to impact their reproductive axis. Finally, fGCM values continued to vary across time, suggesting that the mandrills’ HPA-axes were still responding to stimuli.

The decrease in fGCMs over time in the pre-release enclosure in these rehabilitated mandrills contrasts with findings for wild animals. For example, wild zebra showed little acclimatization to their enclosure over 40 days (Franceschini *et al.,* 2008), and wild eastern bettongs (*Bettongia gaimardi*) did not appear to acclimatize to pre-release enclosures over 95 to 345 days (Batson *et al.,* 2017). While both wild animals and those that are accustomed to life in an enclosure are likely to experience acute stress as a result of transfer, wild animals may be more likely to experience prolonged stress if held captive at a release site. Thus, pre-release enclosures may be more useful for captive animals who have had an opportunity to habituate to the presence of humans and an enclosure, like the mandrills in our study, than for wild animals. These differences across studies highlight the importance of measuring animal welfare as a part of the translocation process in each study species and taking individual history into account.

### After Release

Release into the forest was not correlated with a significant increase in fGCMs in comparison to the final levels in the pre-release enclosure (contra Hypothesis 3, Table 1). This suggests that the release did not produce a further increase in the stress response in the mandrills overall. This is a positive, and rather surprising result, because the unfamiliar challenges of life in the wild might be expected to be stressful for animals used to living in captivity (Parker *et al.,* 2015) and suggests that the pre-release enclosure was useful in providing a safe space while the mandrills adjusted to the forest environment. The continued presence of their groupmates and human observers, combined with ongoing provisioning and access to the enclosure (Groups 1 and 2), may have helped with this transition. However, individual responses varied widely, and fGCM values in two males tripled during the first week after release.

After release, fGCMs continued to decrease over time, suggesting that the mandrills continued to acclimatize to their new environment (Hypothesis 4, Table 1). This decrease in fGCM values occurred despite an increase in physical activity after release, which would be expected to increase glucocorticoid levels. fGCMs vary with season in mandrills (Setchell *et al.,* 2008; Charpentier *et al.,* 2018), but this does not explain the patterns we observed, because the mandrills were released in different seasons, and season would not explain the decreases in fGCMs we observed over time.

The decrease over time suggests that, overall, the mandrills adjusted to their new environment. As in the pre-release enclosure, the decrease may be a result of reduced physiological response to stressors due to prolonged exposure, rather than to reduced stress (Cyr and Romero, 2009). However, again, the continued within-individual variation in fGCM values in the mandrills does not suggest a reduced response to stressors. Moreover, observations of ovarian cycling and pregnancy in females (**Online Resource D**) and two successful births following release suggest that the animals were not physiologically suppressed due to chronic stress.

fGCM levels after release decreased below final sanctuary levels after just over a month and were about half those in the sanctuary 1 year after release. Data for wild mandrills using the same assay would be very useful to put these findings into context (e.g. Pinter-Wollman *et al.,* 2009), but final values lower than the captive initial environment, along with continuing intra-individual variation, suggest that the mandrills adjusted well to the forest environment after release. These patterns for mandrills are similar to those for wild mantled howler monkeys, which showed fGCM levels below pre-capture levels 1 to 4 weeks after translocation from a degraded forest (Aguilar-Cucurachi *et al.,* 2019). fGCM levels in the translocated group of wild zebras returned to pre-capture levels after release, but took longer (11–18 weeks, Franceschini *et al.,* 2008). In contrast, fGCMs had not returned to levels before capture 6 months after translocation in woylies (*Bettongia penicillata*) (Hing *et al.,* 2017). Cross-population comparisons suggest fGCMs continue to decrease over up to 24 years in translocated wild elephants (*Loxodonta africana*) (Jachowski *et al.,* 2013), suggesting that physiological acclimatization can take a very long time in long-lived species*.* A follow-up study of the translocated mandrills would be very useful to test for long-term behavioral and physiological adaptation after translocation.

### Translocation and Animal Well-Being

Many primate releases are conducted based on the assumption that it improves animal welfare (Beck, 2018). However, this is an empirical question. When we compared fGCM values across living conditions, post-release values were lower, on average, than those in the sanctuary or the pre-release enclosure (using values adjusted for the effect of drying time). This pattern suggests that, overall, the release environment was less physiologically challenging to the mandrills than that of the sanctuary or the pre-release enclosure (Hypothesis 5, Table 1). However, although statistically significant, the overall changes are small (1.04-fold increase from the sanctuary to the pre-release enclosure, 0.82-fold decrease from the pre-release enclosure to post-release, and 0.86-fold decrease between the sanctuary and post-release). These differences are smaller than the effects of pregnancy in females, mating and rank instability in males or season. Pregnancy increased fGCMs 1.20-fold relative to cycling females in semi-free ranging mandrills (Setchell *et al.,* 2008) and 1.27-fold relative to other mature females in the descendants of the mandrills released into Lékédi Park (Charpentier *et al.,* 2018). Semi-free ranging males experienced a 1.17-fold increase in fGCMs in months with mating relative to months without mating, and a 1.11-fold increase when male ranks were unstable relative to when they were stable (Setchell *et al.,* 2010). Finally, fGCM concentrations peaked during the short dry season in mandrills at Lékédi Park, with a 1.34-fold increase compared to the short rainy season, when the lowest fGCM values occurred, although much of this difference was due to the presence of pregnant females in the short dry season (Charpentier *et al.,* 2018).

Our overall comparisons between living conditions obscure shorter-term variation in fGCMs. For example, the initial 1.93-fold increase in fGCMs during the first week after transfer to the pre-release enclosure (vs. the final month at the sanctuary) was greater than the overall comparison between the sanctuary and the pre-release enclosure (1.04-fold increase). This difference is partly explained by decreases in fGCM values over time in the pre-release enclosure, and partly by lower fGCM values during the last month at the sanctuary than the overall mean values for the sanctuary. The explanation for the latter difference is unclear, but it is possible that staff paid particular attention to animal welfare before each transfer, or that social relationships among the group were more stable at these times than at other times. Nonetheless, the large increase seems to have been a short-term response to transfer, which then decreased, suggesting that the effect on the mandrills was also short-term. The animals appeared healthy subsequently, suggesting that any costs incurred were manageable. The same applies to the short-term peaks in fGCMs after release in some animals.

Together, comparisons of the magnitude of changes in fGCMs related to the translocation process with seasonal and life history changes in mandrill fGCMs suggest that the overall effect of the translocation steps may be within their normal reactive scope (Romero *et al.,* 2009) and that any spikes into homeostatic overload were short-term. If this is the case, the mandrills may have been able to respond to the challenges of the translocation with few deleterious effects on their health. However, in common with other studies (Romero *et al.,* 2015), we do not know where the boundary between the normal reactive scope and homeostatic overload falls (and it will vary among individuals), making it difficult to draw conclusions about whether the translocation moved individuals into homeostatic overload, or for how long. Moreover, maintaining physiological systems in the reactive homeostasis range is costly, the cost increases with time spent in this range and a gradual decrease in the ability to cope means that individuals eventually move into homeostatic overload (Romero *et al.,* 2009). In other words, even if the mandrills coped with the translocation, they may still have paid a physiological cost.

The overall magnitude of changes in fGCMs across living conditions in this study are also mild in comparison to those for translocated wild mantled howler monkeys (Aguilar-Cucurachi *et al.,* 2019). In the howlers, fecal corticosterone increased 1.08-fold when the animals were captured from the wild and held in a social enclosure for approximately 2 months, increased a further 1.32-fold when they were moved to an outdoor 0.18 ha enclosure for one month, then decreased 0.57-fold in the month after they were released into a protected forest. Values after release were 0.81-fold lower than in the initial environment. These patterns suggest that the howlers’ experience of translocation was more challenging than that of the mandrills, and that translocation led to a greater improvement in their well-being. This difference may be a species difference but is also likely to be because the translocated howlers were wild, while the mandrills were habituated to enclosures at the sanctuary. The large decrease in fGCMs from the howlers’ initial environment, in a degraded forest environment, and the final protected forest environment suggests that the initial environment was also physiologically challenging, as shown by their poor condition at the start of the study (Aguilar-Cucurachi *et al.,* 2019)*.* In contrast, the mandrills appeared to be in good physical condition when housed at the sanctuary, and the overall decrease in fGCM levels was smaller in the mandrills than in the howlers. These differences underline the need to consider the source of the animals when planning a translocation, as highlighted by the IUCN/SSCC reintroduction guidelines (IUCN/SSC, 2013)*.*

## Conclusions

Many translocation programs do not monitor the animals post-release sufficiently to assess the outcome, or use popular media to promote positive news, rather than reporting full outcomes scientifically (Beck, 2018). Physiological stress is an unavoidable aspect of translocation and may impair an animal's chance of survival, so measures should be taken to minimize the stress animals experience during translocation (Teixeira *et al.,* 2007; Dickens *et al.,* 2010). Our study suggests that a soft release, with a pre-release enclosure and support post-release including food, observations of the animals’ condition and interventions to remove or rescue animals, can work for wild-born, orphaned mandrills who have been rescued and rehabilitated in a sanctuary. Measuring fGCMs when the mandrills were housed in the sanctuary, in a pre-release enclosure and after release into their natural habitat provided insights into the benefit of using a pre-release enclosure to reduce the potentially cumulative effects of stress in translocations. Overall, our findings suggest that the translocation process did not have detrimental effects on the mandrills, and that release to wild may have improved their well-being compared to sanctuary housing. Our study illustrates the contribution of non-invasive monitoring of animal physiology to the scientific evaluation of animal translocations, protocol design and, ultimately, to translocation success.

## Funding Statement

This work was supported by the Jane Goodall Institute and private donors.

## Author Contributions Statement

M.C.W. participated in the conceptualization, methodology, investigation—fieldwork, formal analysis, writing—original draft, project administration. R.A. and D.C. participated in the resources and project administration. G.T.W. participated in the investigation—fieldwork. C.J.W. participated in the methodology. investigation—laboratory analysis, writing—review and editing, visualization. S.R.L. participated in the methodology, investigation—laboratory analysis, data curation, visualization. J.M.S.participated in the supervision, conceptualization, methodology, formal analysis, data curation, writing—original draft, writing—review and editing, visualization.

## Conflicts of Interest Statement

The authors report no conflict of interest.

## Data Availability Statement

The data that support the findings of this study are openly in the online supplementary material and in figshare at 10.6084/m9.figshare.22591807.

## Supplementary Material

Web_Material_coad025
