# Supplementary material for: Successful acclimatization of mandrills (Mandrillus sphinx) translocated to Conkouati-Douli National Park, Republic of Congo, as measured by fecal glucocorticoid metabolites
Source: Conserv Physiol. 2023 Jun 8;11(1):coad025. doi: 10.1093/conphys/coad025 (PMC10170324; doi:10.1093/conphys/coad025)
Supplement: Web_Material_coad025 [file web_material_coad025.zip › Online appendices A-D with new photo.pdf]

Online appendices for Woodruff et al ‘Successful acclimatization of mandrills (*Mandrillus sphinx*) translocated to Conkouati-Douli National Park, Republic of Congo, as measured by fecal glucocorticoid metabolites’

Appendix A: Supplementary Methods, Figs S1-3

Mandrill enclosures at Tchimpounga Sanctuary

Each enclosure was divided into two sections by a chain link fence above a 1 m brick foundation and a sliding door. The enclosures had diagonal structural elements passing through the center and fire hoses or hammocks as enrichment, and platforms in the corners to allow the mandrills to leave the ground.

The three sections of the enclosure had a total of ~58 m^2 of covered space. Construction of the third section was completed after Group 1 was transferred to the release site. We included two chain link outdoor runs without grooves to allow the mandrills to become accustomed to looking above them for food and predators and to foraging in the leaf litter

These enclosures were constructed to establish a long-term project. For temporary projects that aim to leave no permanent footprint, a strong chain-link fence, metal sheeting for the roof, a water supply and posts made of wood treated for insect resistance would likely be sufficient for a pre-release enclosure. This would greatly reduce material, transport, and labor costs.

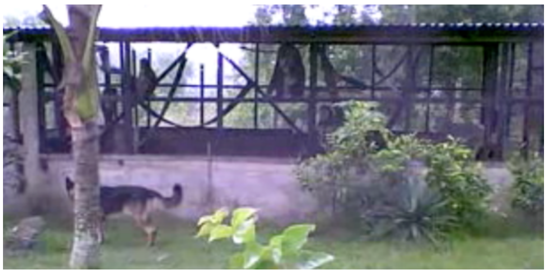

Front view far left enclosure

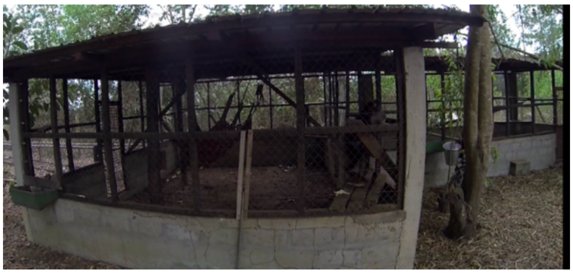

Front view far right enclosure

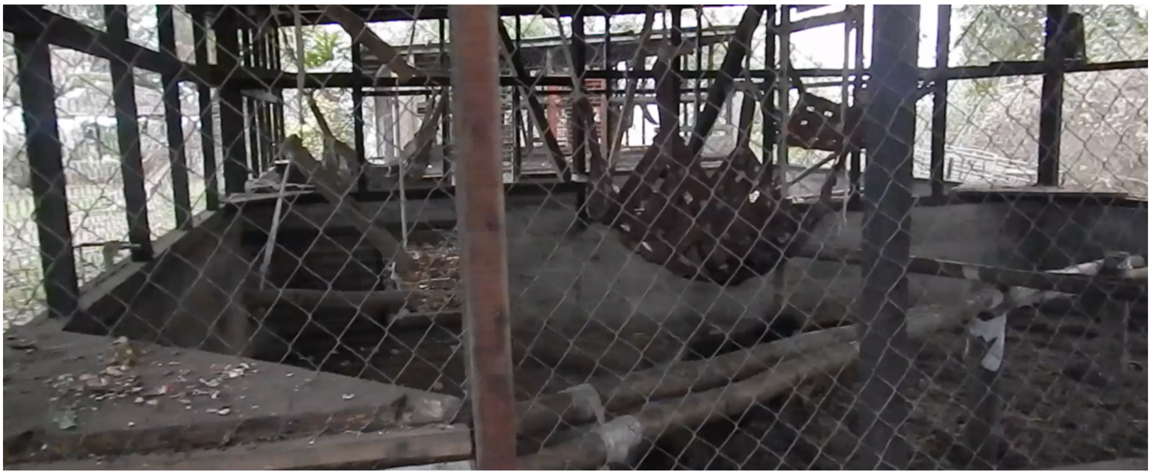

Corner platforms, swings, hammocks, and bamboo enrichment structures

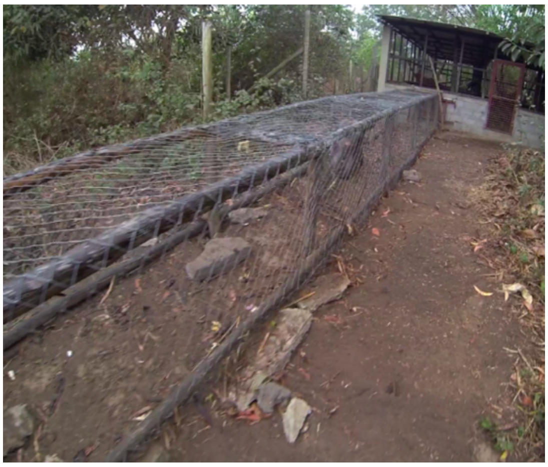

Chain link corridor linking mandrill enclosures 1 and 3

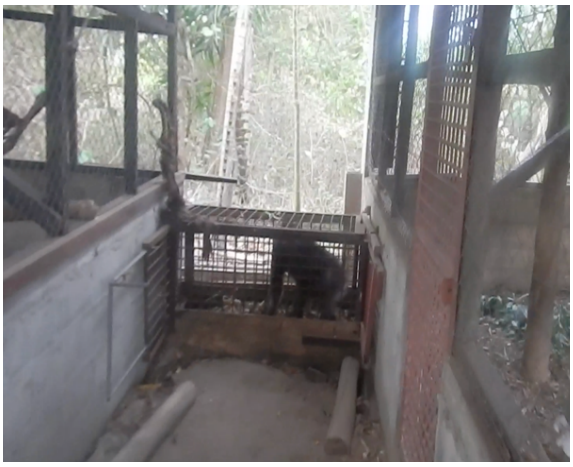

Permanent connecting corridor between enclosure 2 and 3

Figure S1: The mandrill enclosures at Tchimpounga Sanctuary, Tchimpounga Reserve, Republic of Congo, 2013-15.

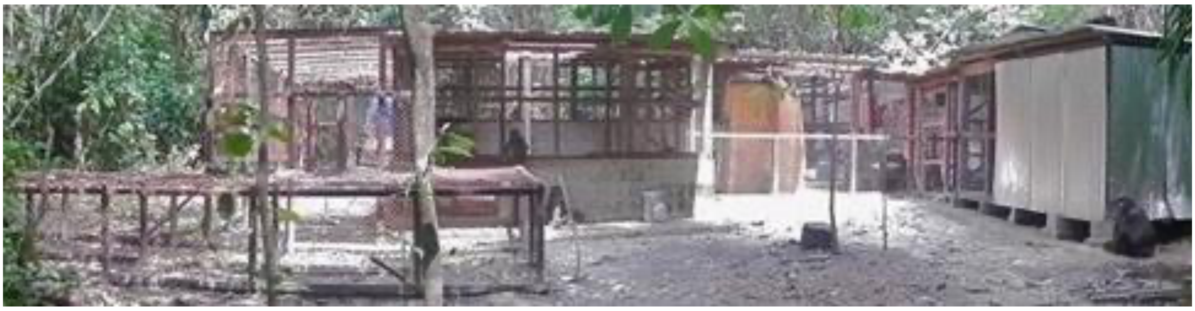

View of the pre-release compound

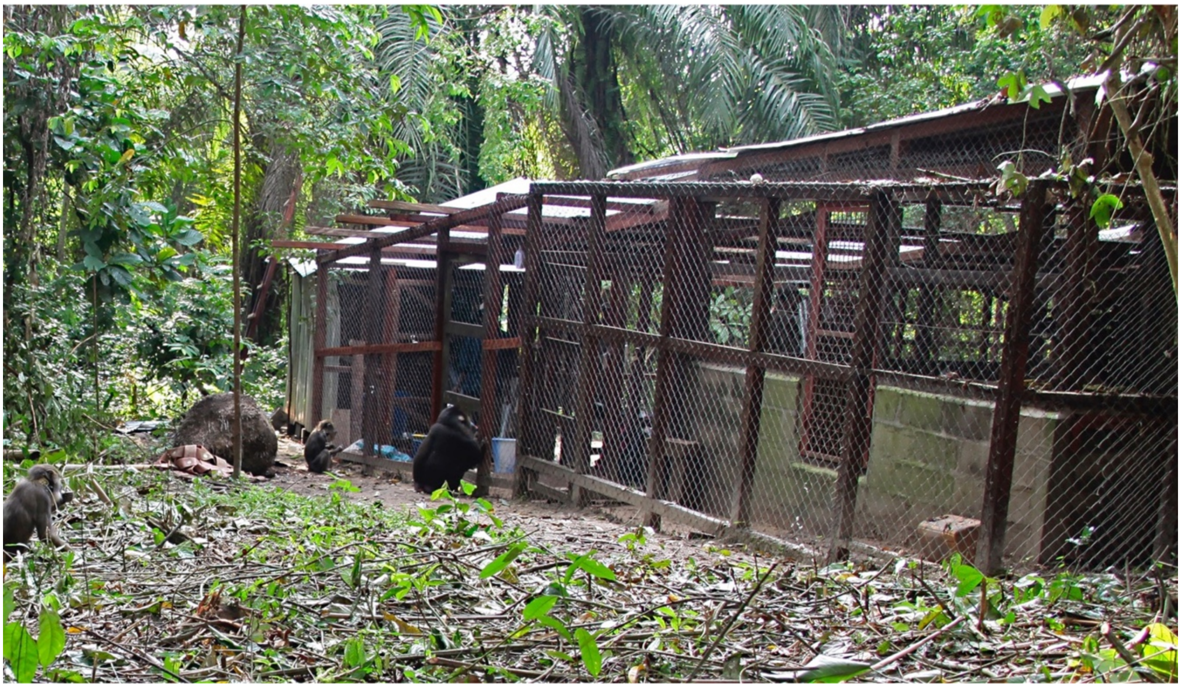

Staff housing (furthest from photographer), food preparation area (middle), and the pre-release enclosure (closest to the photographer).

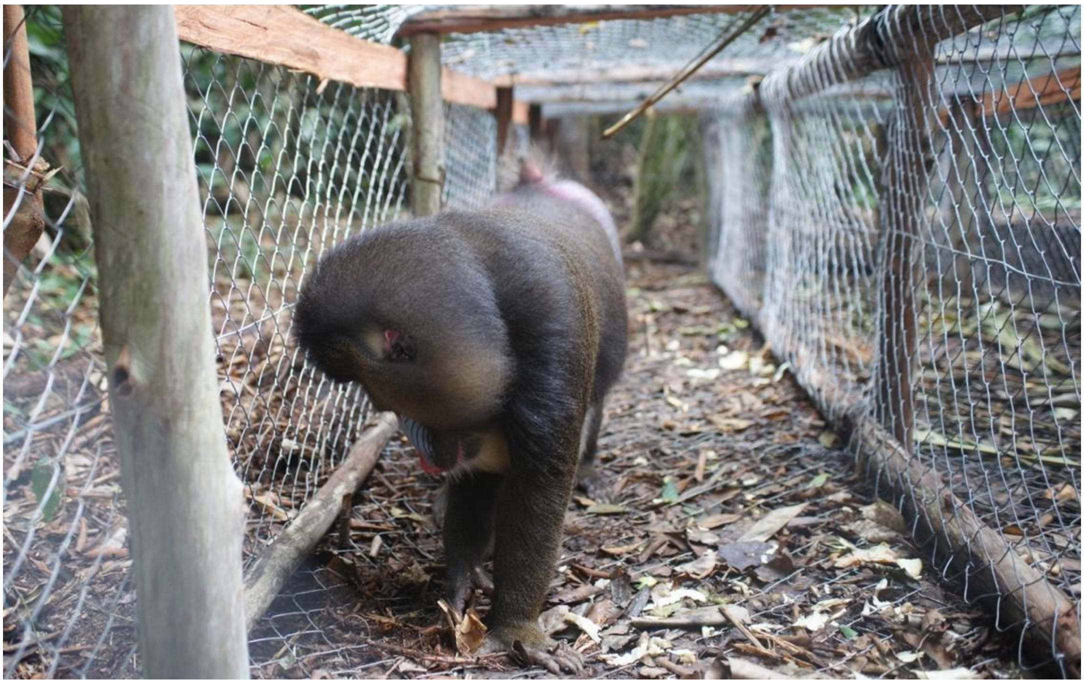

Outdoor corridor

Figure S2: The pre-release compound for a release of mandrills in Conkouati-Douli National Park, Republic of Congo, 2013-15.

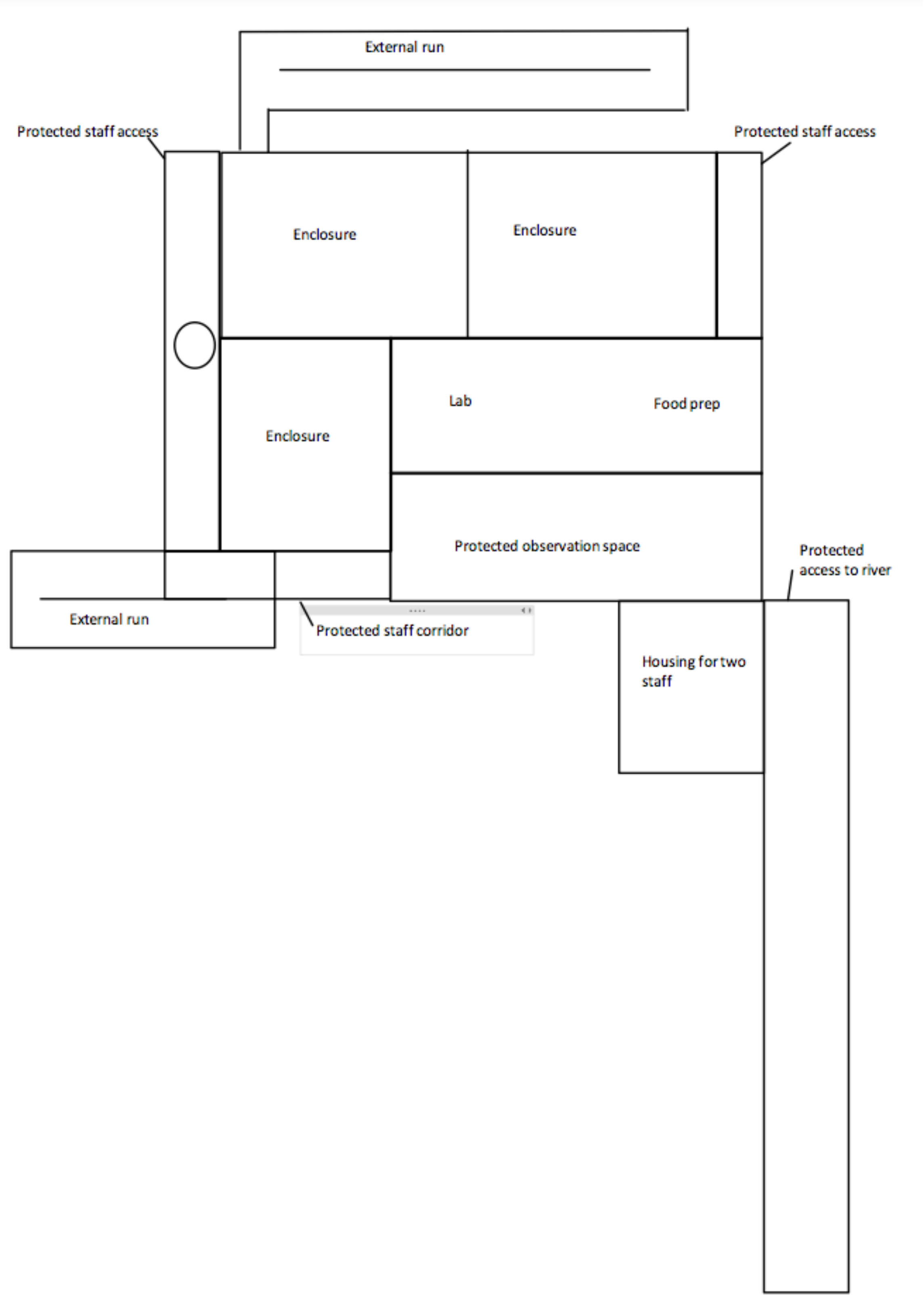

Figure S3: Plan of the pre-release compound for a release of mandrills in Conkouati-Douli National Park, Republic of Congo, 2013-15 (exact scale not available, but the three mandrill enclosures combined are 58 m^2)

Appendix B: Supplementary Results, Table S1, Figures S4-6

Table S1: Results of General Linear Mixed Models testing predictions 1 and 5 related to fecal glucocorticoid values in mandrills released into Conkouati-Douli National Park, Republic of Congo, 2013-15. Sanctuary values not adjusted to account for the effect of faster drying time on fGCM values.

Prediction	Parameter	Estimate	SE	df	t	p	95% CI lower	95% CI upper
1	Intercept	2.855	0.133	83	21.485	<0.001	2.591	3.210
	Sanctuary vs. pre-release enclosure[1]	-0.423	0.051	70.688	-8.29	**<0.001**	-0.524	-0.321
	Female vs male[1]	0.062	0.067	6.06	0.92	0.391	-0.101	0.225
2 (first 30 days)	Intercept	2.868	0.129	141	22.27	<0.001	2.613	3.123
	Days since transfer to pre-release enclosure	-0.010	0.002	131.11	-4.83	**<0.001**	-0.014	-0.006
	Female vs male[1]	0.081	0.073	11.75	1.12	0.286	-0.077	0.240
2 (after 30 days)	Intercept	2.714	0.189	202	14.37	<0.001	2.341	3.086
	Days since transfer to pre-release enclosure	0.000	0.000	190.06	-0.74	0.462	-0.001	0.001
	Female vs male[1]	-0.049	0.081	5.92	-0.60	0.570	-0.248	0.150
5	Intercept	2.602	0.163	982	15.99	<0.001	2.282	2.921
	Sanctuary vs. forest[1]	-0.070	0.029	901.47	-2.43	**0.015**	-0.126	-0.013
	Pre-release enclosure vs. forest[1]	0.084	0.020	963.06	4.21	**<0.001**	0.045	0.123
	Female vs male[1]	0.056	0.065	11.70	0.876	0.399	-0.084	0.196

[1]Reference group

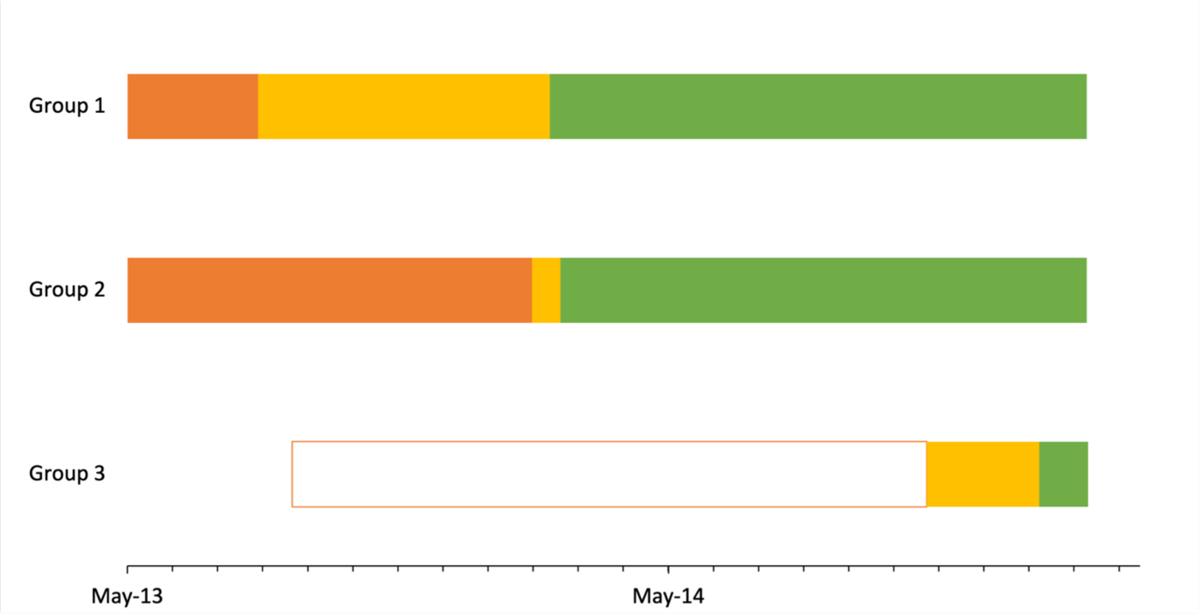

Figure S4: Timeline for a mandrill translocation into Conkouati-Douli National Park, Republic of Congo, 2013-15. Orange indicates time at the sanctuary, yellow indicates time in the pre-release enclosure, green indicates time in the forest. Groups 1 and 2 were sampled in each living condition. Group 3 were not sampled while housed at the sanctuary because staff were busy elsewhere (indicated in the timeline as no fill). Groups 1 and 2 were housed at the sanctuary before the start of the study in May 2013. Animals in Group 3 arrived at the sanctuary between Sept 2013 and April 2014 (the bar begins with the earliest arrival).

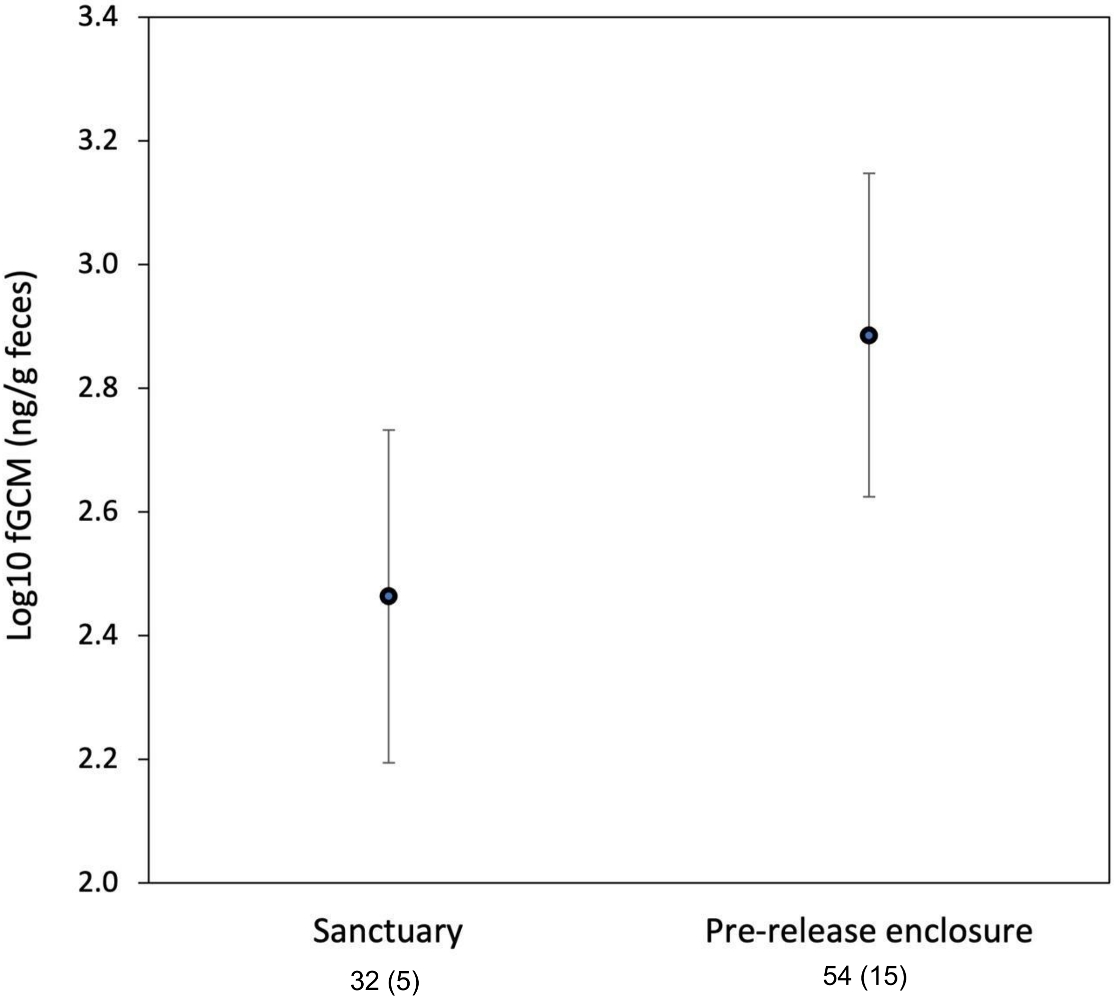

Figure S5: Estimated marginal means with 95% CI fecal glucocorticoid metabolite (fGCM) values in mandrills during their last month in Tchimpounga Sanctuary and their first week in a pre-release enclosure in Conkouati-Douli National Park, Republic of Congo, in 2014-15. Numbers below x axis show number of samples (number of animals). Sanctuary values not adjusted for the effect of differences in drying time.

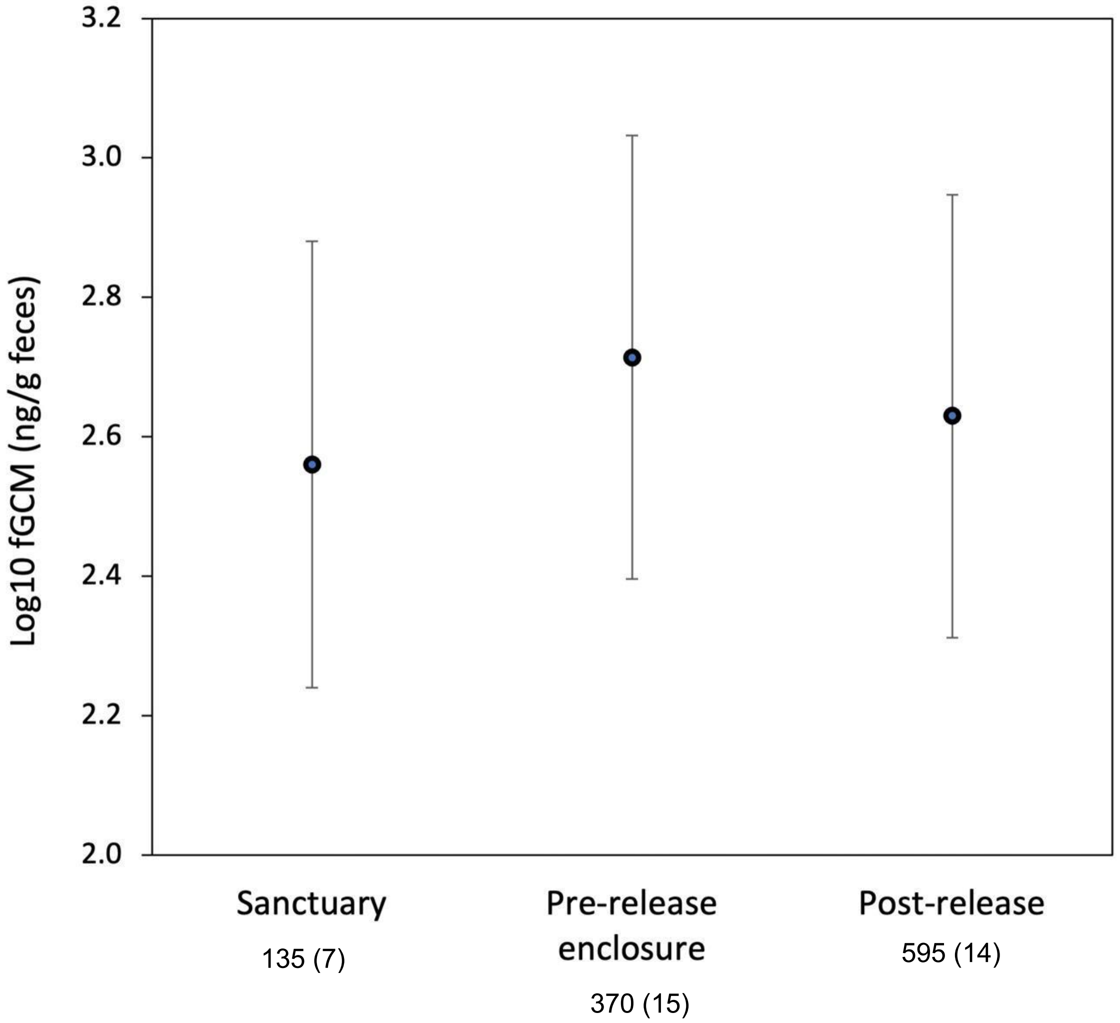

Figure S6: Estimated marginal means with 95% CI fecal glucocorticoid metabolite (fGCM) levels in mandrills housed in Tchimpounga sanctuary, a pre-release enclosure, and after release into Conkouati-Douli National Park, Republic of Congo, in 2014-15. Numbers below y axis are number of samples (number of animals). Sanctuary values not adjusted to account for the effect of differences in drying time (see methods).

Appendix C: Supplementary Discussion

Mandrill translocation strategies

To our knowledge, two projects have released mandrills into the wild. In 2002, the Centre International de Recherches Médicales de Franceville (CIRMF), Gabon, released 36 mandrills into a very large (1 750 ha) forested enclosure in Lékédi Park, Gabon, with 33% mortality in the first 8 weeks of the 12 month follow-up (Peignot et al., 2008). The animals spent 12-34 days in a pre-release enclosure but left the release area within hours, despite the availability of supplemental food (Peignot et al., 2008). Their departure may have been related to a lack of shelter in the pre-release enclosure, which was in a savanna, with only a roofed cage and a few small bushes for shade. The animals were visibly stressed in the enclosure (Setchell, pers obs), and may have run to the forest to seek shade. The exact causes of the deaths in this release are unknown, because the animals were tracked at a distance using radio collar triangulation for the first 8 weeks, but the remaining animals were thin when they were observed after 8 weeks. The authors attributed the deaths to environmental stress and malnutrition and instituted supplemental feeding for at least 2 years. Survival improved the following year, and reproduction occurred. The authors recommend avoiding hard releases of mandrills, where animals do not receive training or support before or after the release (Kleiman, 1989), and not releasing pregnant females or those with dependent offspring (Peignot et al., 2008). CIRMF have since released a further group or groups of mandrills into the same park, but these releases are undocumented.

The other project to have released mandrills into the wild is the Jane Goodall Institute (JGI) Tchimpounga Chimpanzee Rehabilitation Center (Tchimpounga), who released a group or groups (records unclear) of 1-4 mandrills into Conkouati-Douli National Park, Congo, in 2009. They transferred the mandrills to a release site in pet carriers and released them in an area where supplemental food was available. The mandrills left the release site within hours or days and were not seen again. No information is available on the exact timing or methods used in the releases, or on the animals' behavior.

Building on these experiences in a new release project, Tchimpounga decided to build a pre-release enclosure at the release site to allow mandrills to acclimatize to the forest before release. This created the opportunity to study the animals for an extended period in three phases of the release process: at the sanctuary, in the pre-release enclosure, and post-release.

Of 15 mandrills chosen for translocation, 12 were translocated successfully and survived to the end of the study, with two successful births. Three animals were returned to the sanctuary due to behavioral concerns. We attribute the overall success of this translocation (within the duration of the study) to careful planning, extensive (and expensive) post-release monitoring, and provision of a great deal of support for the released animals. However, the translocation was logistically complicated and expensive, and translocation cannot be regarded as a cheap option for dealing with surplus animals.

The release strategy for these mandrills was heavily influenced by methods used for chimpanzees (*Pan troglodytes*) and included splitting the original stable group into subgroups for release. This disrupted the group and caused conflict when sub-groups were reunited. Keeping the group together through the release process and then releasing them at the same time may be a better strategy for species with strong social bonds and low fission-fusion dynamics. Nevertheless, the three release groups merged successfully. Groups 1 and 2 had previously formed a single group and settled quickly when they were reunited,

after initial conflict between some individuals. Group 3, in contrast, comprised five juveniles unfamiliar with Groups 1 and 2. Mandrill group sizes range from fewer than 50 to hundreds in the wild (Rogers et al., 1996; Abernethy et al., 2002; Hongo, 2014), much larger than the overall group size in this translocation, with possible consequences for the animals' long-term survival. Our experience of adding new members to the group suggests that it may be possible to reinforce the group in future.

Our study suggests that an extended stay in a pre-release enclosure in the forest, combined with careful post-release attention, reduces the likelihood that mandrills travel far from the release area. In contrast to the previous releases, the mandrills in our study remained in the forested release area, allowing us to combine sub-groups, and all but one mandrill stayed with the group during the post-release monitoring.

The one mandrill who left the group during our study was an adolescent male. This male spent only 21 days in the pre-release enclosure prior to release and received high levels of aggression from other group members, particularly when the group was reunited after being split up for release. Adolescent and adult males were also more likely to leave the group In the CIRMF release (Peignot et al., 2008). This is in line with what we know about male mandrills, who begin to become peripheral to their natal group around the age of 6 years (Setchell et al., 2006). Some adult males are also peripheral to groups or solitary (Wickings and Dixson, 1992). Because provisioning is required post-release (Peignot et al., 2008) and adolescent and adult males are more likely to disperse, they may be less suitable for release than juvenile males and females of any age. This argues for releasing males as juveniles, if possible, to give them time to adapt to their new environment before they mature.

References

Abernethy KA, White LJT, Wickings EJ. 2002. Hordes of mandrills (*Mandrillus sphinx*): extreme group size and seasonal male presence. J Zool 258:131–137. http://doi.wiley.com/10.1017/S0952836902001267

Hongo S. 2014. New evidence from observations of progressions of mandrills (*Mandrillus sphinx*): a multilevel or non-nested society? Primates 55:473-481. https://doi.org/10.1007/s10329-014-0438-y

Kleiman DG. 1989. Reintroduction of captive mammals for conservation: Guidelines for reintroducing endangered species into the wild. Bioscience 39:152–161. https://doi.org/10.2307/1311025

Peignot P, Charpentier MJE, Bout N, Bourry O, Massima U, Dosimont O, Terramorsi R, Wickings EJ. 2008. Learning from the first release project of captive-bred mandrills *Mandrillus sphinx* in Gabon. Oryx 42:122–131. https://doi.org/10.1017/S0030605308000136

Rogers ME, Abernethy KA, Fontaine B, Wickings EJ, White LJT, Tutin CEG. 1996. Ten days in the life of a mandrill horde in the Lope Reserve, Gabon. Am J Primatol 40:297–313. https://doi.org/10.1002/(SICI)1098-2345(1996)40:4%3C297::AID-AJP1%3E3.0.CO;2-T

Setchell JM, Wickings EJ, Knapp LA. 2006. Life history in male mandrills (*Mandrillus sphinx*): Physical development, dominance rank, and group association. Am J Phys Anthropol 131:498-510. doi: 10.1002/ajpa.20478.

Wickings EJ, Dixson AF. 1992. Testicular function, secondary sexual development, and social status in male mandrills (*Mandrillus sphinx*). Physiol Behav 52:909–916. https://doi.org/10.1016/0031-9384(92)90370-H

Appendix D: Supplementary Discussion

Ovarian cycling in female mandrills translocated from Tchimpounga Sanctuary to Conkouati-Douli National Park, Republic of Congo, in 2013-15

We provide fecal progesterone for three females for whom we had sufficient samples, and plot this with sexual swelling scores to assess ovarian cycling.

Field methods

We scored female sexual swellings opportunistically using the scoring system used at CIRMF, Gabon:

0: flat swelling 0 to ~0.5 inches. Includes the small pregnancy swelling.

1: increasing from 0 to 1/3 of its full volume or decreasing from 1/3 of its full volume to flat.

2: increasing to 1/3 to 2/3 of its full volume or decreasing from 2/3 to 1/3 of its full volume.

3: increasing to 2/3 of its full volume to full volume where the skin is taut and shiny or decreasing from its full volume to 2/3 of its volume.

Laboratory methods

We used the same extracted fecal samples for progesterone as for glucocorticoid metabolites (see Methods). We used an enzyme immunoassay described previously (Graham et al. 2001) based on a monoclonal antibody CL425 produced against 4-pregnen-11-ol-3,20-dione hemisuccinate: BSA (J. Roser, University of California-Davis).

P4 concentrations in a serial dilution of mandrill fecal extracts were parallel to the standard curve, based on visual inspection and a linear regression of the percent binding of the labeled progesterone in the P4 assay including the interaction of the log of the standard concentration or log of the inverse of the sample dilution * category of standard versus sample (interaction term $P>0.05$ indicating that the lines do not intersect; Systat 13).

We diluted samples 1:130 in assay buffer.

Coefficients of variation for intra-assay replicates were <20%, and inter-assay controls (n = 4 96-well plates) were 2% and 10% for the high and low concentrations, respectively. Assay sensitivity was 3.22 pg/well (90% binding).

Results

Ovarian activity was apparent upon inspection of fecal P4 concentrations and swelling scores. Sustained elevated progesterone concentrations were consistent with pregnancy (MOB and DOM).

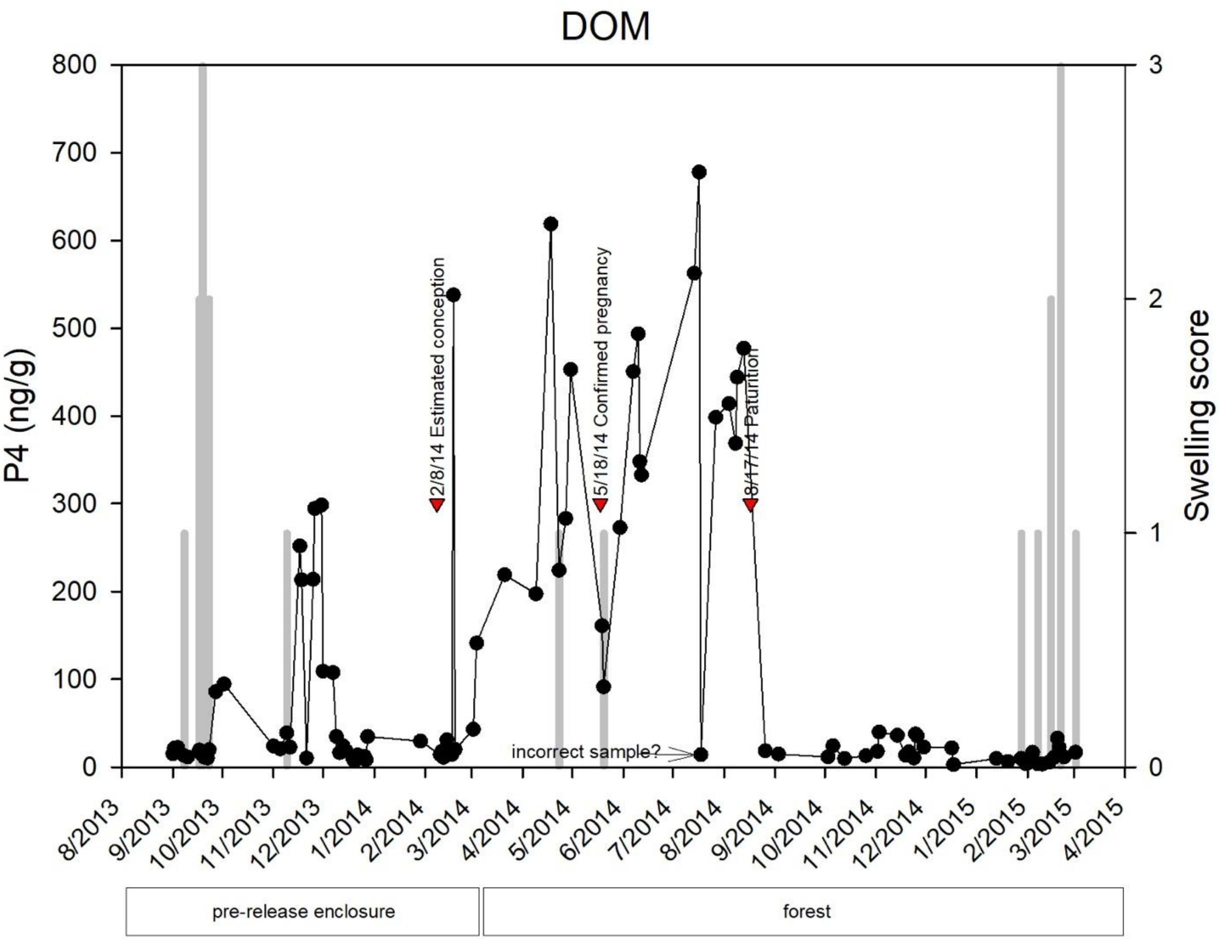
DOM
P4 (ng/g)
Swelling score
2/8/14 Estimated conception
5/18/14 Confirmed pregnancy
8/17/14 Paturition
incorrect sample?
8/2013
9/2013
10/2013
11/2013
12/2013
1/2014
2/2014
3/2014
4/2014
5/2014
6/2014
7/2014
8/2014
9/2014
10/2014
11/2014
12/2014
1/2015
2/2015
3/2015
4/2015
pre-release enclosure
forest

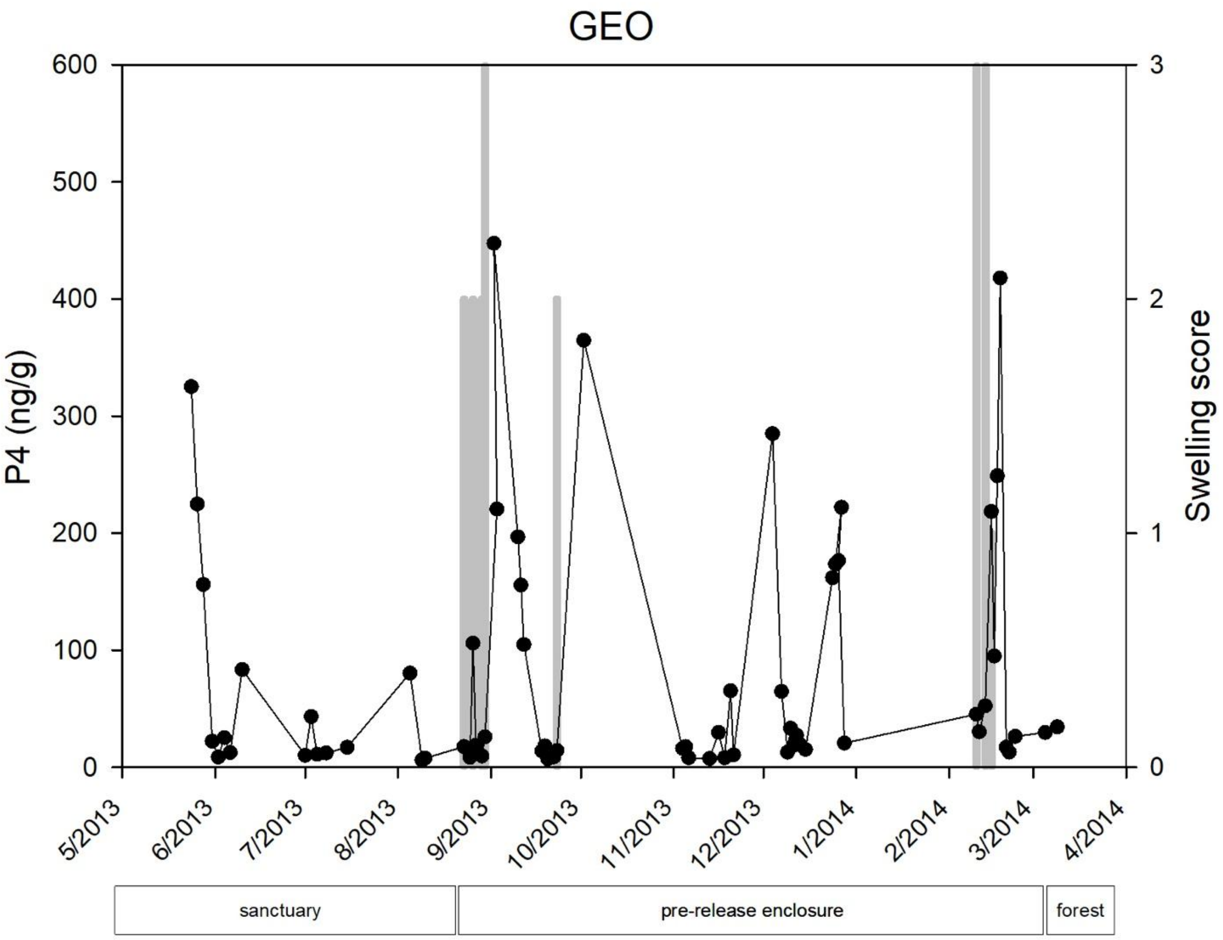

GEO
P4 (ng/g)
Swelling score
0
100
200
300
400
500
600
0
1
2
3
5/2013
6/2013
7/2013
8/2013
9/2013
10/2013
11/2013
12/2013
1/2014
2/2014
3/2014
4/2014
sanctuary
pre-release enclosure
forest

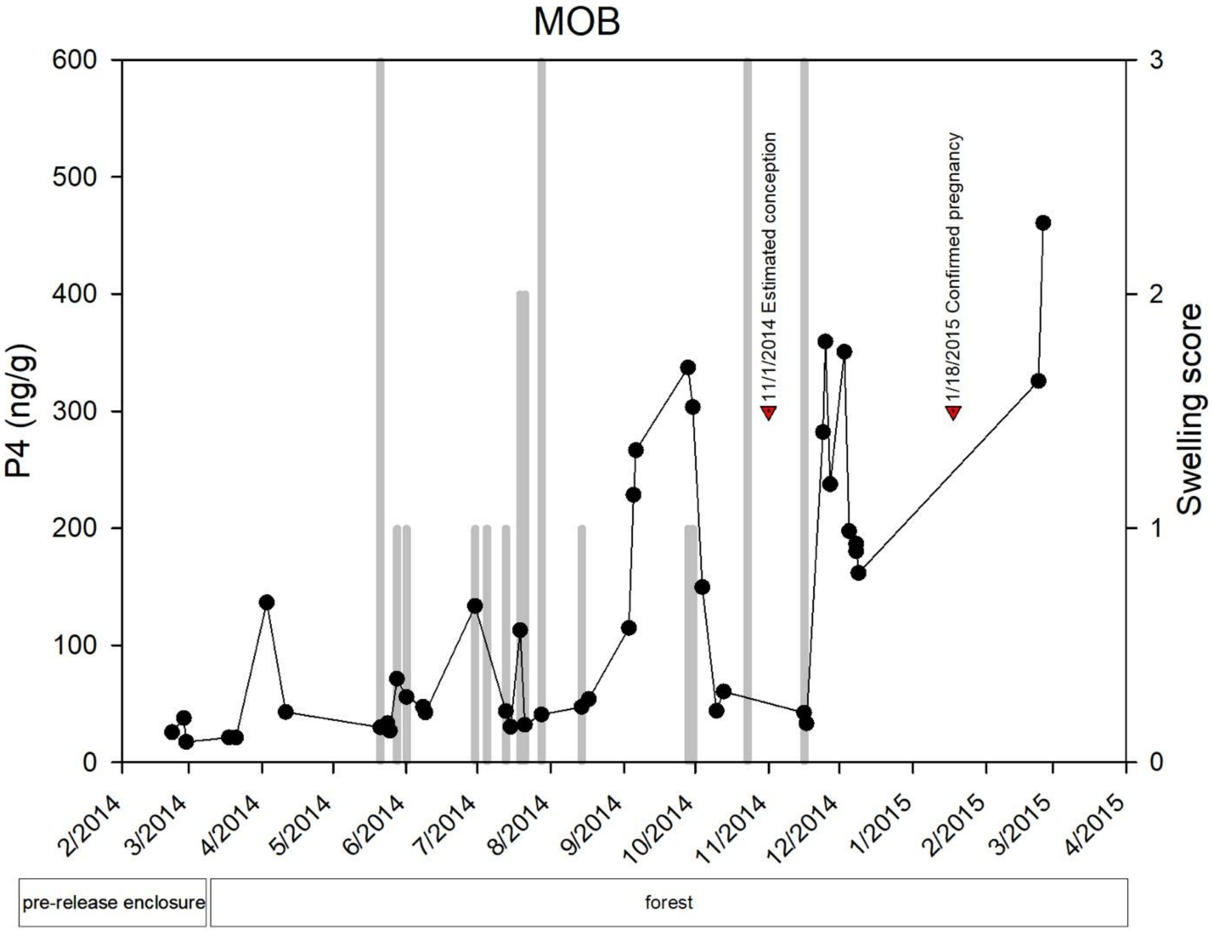

Figure S7: Fecal P4 and sexual swelling scores for three female mandrills during their translocation from Tchimpounga Sanctruary to Conkouati-Douli National Park, Republic of Congo, in 2013-15. Top to bottom: Dominique, George, Mobote.

References

Graham LH, Schwarzenberger F, Mostl E, Galama W, Savage A. 2001. A versatile enzyme immunoassay for the determination of progestogens in feces and serum. Zoo Biol 20:227–36.
